# Children undergoing cardiac surgery for complex cardiac defects show imbalance between pro- and anti-thrombotic activity

**DOI:** 10.1186/cc5108

**Published:** 2006-11-24

**Authors:** Ruth Heying, Wim van Oeveren, Stefanie Wilhelm, Katharina Schumacher, Ralph G Grabitz, Bruno J Messmer, Marie-Christine Seghaye

**Affiliations:** 1 Department of Pediatric Cardiology, University Hospital, RWTH-Aachen, Pauwelsstrasse 30, 52074 Aachen, Germany; 2 Department of BioMedical Engineering, University of Groningen, A. Deusinglaan1, 9713 AV Groningen, The Netherlands; 3 Department of Cardiothoracic and Vascular Surgery, University Hospital, RWTH-Aachen, Pauwelsstrasse 30, 52074 Aachen, Germany

## Abstract

**Introduction:**

Cardiac surgery with cardiopulmonary bypass (CPB) is associated with the activation of inflammatory mediators that possess prothrombotic activity and could cause postoperative haemostatic disorders. This study was conducted to investigate the effect of cardiac surgery on prothrombotic activity in children undergoing cardiac surgery for complex cardiac defects.

**Methods:**

Eighteen children (ages 3 to 163 months) undergoing univentricular palliation with total cavopulmonary connection (TCPC) (*n *= 10) or a biventricular repair (*n *= 8) for complex cardiac defects were studied. Prothrombotic activity was evaluated by measuring plasma levels of prothrombin fragment 1+2 (F1+2), thromboxane B_2 _(TxB2), and monocyte chemoattractant protein-1 (MCP-1). Anti-thrombotic activity was evaluated by measuring levels of tissue factor pathway inhibitor (TFPI) before, during, and after cardiac surgery.

**Results:**

In all patients, cardiac surgery was associated with a significant but transient increase of F1+2, TxB2, TFPI, and MCP-1. Maximal values of F1+2, TxB2, and MCP-1 were found at the end of CPB. In contrast, maximal levels of TFPI were observed at the beginning of CPB. Concentrations of F1+2 at the end of CPB correlated negatively with the minimal oesophageal temperature during CPB. Markers of prothrombotic activity returned to preoperative values from the first postoperative day on. Early postoperative TFPI levels were significantly lower and TxB2 levels significantly higher in patients with TCPC than in those with biventricular repair. Thromboembolic events were not observed.

**Conclusion:**

Our data suggest that children with complex cardiac defects undergoing cardiac surgery show profound but transient imbalance between pro- and anti-thrombotic activity, which could lead to thromboembolic complications. These alterations are more important after TCPC than after biventricular repair but seem to be determined mainly by low antithrombin III.

## Introduction

Cardiac surgery with cardiopulmonary bypass (CPB) is associated with inflammatory and haemostatic alterations that may lead to severe bleeding and/or thromboembolic events [1-3]. Abnormalities of the thrombin pathway play a main role in coagulation and fibrinolysis disorders observed in patients undergoing CPB [2,3]. A main marker for thrombin generation is the cleavage of fragment 1+2 from prothrombin (F1+2) [4]. Thrombin is a major activator of platelets. Its activity is associated with early mortality after coronary artery bypass grafting operations [5]. Platelet activation goes along with the activation of the arachidonic acid pathway in platelets and results in the release of the potent platelet-aggregating agent thromboxane A_2_, which is rapidly converted into the inactive product thromboxane B_2 _(TxB2) [6].

Thrombin leads furthermore to tissue factor pathway inhibitor (TFPI) production [7]. TFPI circulates in plasma as a complex bound to lipoproteins. Approximately 10% of circulating TFPI is bound to platelets and is released upon stimulation with thrombin. TFPI inhibits factor VIIa/TF complex and factor Xa. Consumption of TFPI offers insight in extrinsic coagulation disorders such as disseminated intravascular coagulation [7,8].

**Figure 1:**
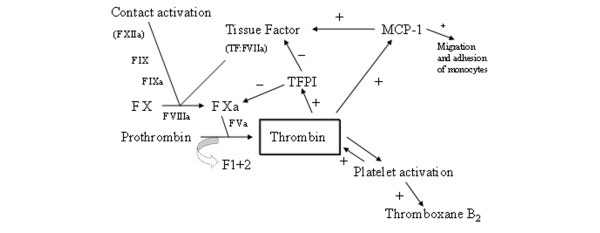
Role of thrombin in the coagulation system. Thrombin has a central role in different interactions of the coagulation system as shown. Cleavage of prothrombin fragment 1+2 (F1+2) from prothrombin results in thrombin formation. Thrombin production leads to tissue factor pathway inhibitor (TFPI) upregulation, which inhibits tissue factor (TF) activity. Monocyte chemoattractant protein-1 (MCP-1) production is also increased via thrombin interaction.

Thrombin also plays a role in inducing the production of monocyte chemoattractant protein-1 (MCP-1) [9]. A wide variety of cells are capable of producing MCP-1, including leucocytes and all cell types of the vascular wall. MCP-1 is a chemotactic factor for monocytes which also stimulates monocyte degranulation, respiratory burst, and tissue factor expression. Figure 1 shows the main role of thrombin in the haemostatic system.

In children with single-ventricle physiology, total cavopulmonary connection (TCPC) is largely accepted as definitive palliation [10,11]. However, besides long-term complications, patients after TCPC are at high risk for thromboembolic events due to the non-pulsatile perfusion of the lungs [12-14]. This study was designed to investigate the influence of cardiac surgery on the balance between pro- and anti-thrombotic activity in children with complex cardiac defects and to address the role of univentricular palliation.

## Materials and methods

### Patients

This study was approved by the Ethical Medical Committee of the Aachen University of Technology, and written informed parental consent was obtained.

#### Clinical data

Eighteen infants and children (ages 3 to 163 months) with complex cyanotic cardiac defect who were scheduled for cardiac surgery were enrolled in this prospective study. Patients were divided into two groups according to the planned operation: TCPC (*n *= 10) and biventricular (BV) repair (*n *= 8). Genetic, inflammatory, or metabolic diseases were excluded in all cases. Epidemiological and clinical patient data, including preoperative medication, are summarised in Table 1.

#### Anaesthesia

Conventional general anaesthesia consisted of diazepam, fentanyl sulphate, and pancuronium bromide. After induction of anaesthesia, nasotracheal intubation was performed, and central venous and peripheral arterial catheters were inserted. Perioperative antibiotic prophylaxis consisted of cefotiam hydrochloride.

#### Surgical procedure and CPB

The CPB protocol was uniform for all children and was performed with a roller pump, a hollow-fibre membrane oxygenator, and an arterial filter. Anticoagulation was achieved by heparin sulphate (3 mg/kg body weight) to reach an activated clotting time (ACT) of more than 400 seconds. Cooling and rewarming were performed with a heat exchanger. The priming solution consisted of a crystalloid solution and mannitol (3 ml/kg). For vasodilatation during the cooling and rewarming periods, a continuous infusion of sodium nitroprusside was given. CPB was instituted with a perfusion index of 2.7 litres/minute per square metre of body surface area. Dexamethasone was given in a dosage of 3 mg/m^2 ^of body surface area. The aorta was cross-clamped, and cardioplegia was induced by a single intra-aortic injection of a 4°C cold Bretschneider solution (30 ml/kg). Cardiocirculatory arrest was instituted if necessary, and the surgical procedure was continued under low-flow perfusion (25% of the calculated initial perfusion rate). Rewarming was achieved under full-flow conditions. The lungs of the patients were reventilated when core temperature reached 30°C. Neutralisation of heparin was achieved with protamine sulphate in a 1:1 ratio. During bypass, 14 children received red blood cell transfusion and 16 children fresh frozen plasma.

#### Postoperative care

Postoperative monitoring included continuous registration of heart rate and rhythm, arterial blood pressure, central venous pressure, and diuresis. Inotropic support consisted in all cases of dopamine and, if necessary, epinephrine and dobutamine. Vasodilatatory treatment with sodium nitroprusside was given in all cases. Diuretics (furosemide, single dosage 0.5 to 1 mg/kg) were administered according to the clinical requirement. All patients received volume substitution up to four hours postoperatively and 16 patients up to 24 hours postoperatively, which consisted mainly of blood transfusions, fresh frozen plasma, or human albumin 5%. There was no significant difference in postoperative volume substitution between the two patient groups. Thrombotic events were defined as thrombus formation in veins, arteries, or cavities of the heart. They were assessed/excluded by clinical examination, vessel sonography, and echocardiography.

Data are shown in Table 2.

### Methods

#### Laboratory tests

Blood samples were taken at 10 different time intervals before, during, and after cardiac surgery as follows: preoperatively, after heparin administration, 10 minutes after onset of CPB, end of CPB, five minutes after protamine administration, four hours postoperatively, 24 hours postoperatively, 48 hours postoperatively, 72 hours postoperatively, and at discharge between postoperative days 10 and 14. Venous blood was collected before and after the operation. During CPB, blood was withdrawn from the arterial line of the circuit. Each sample consisted of 2 ml of blood anticoagulated with ethylenediaminetetraacetic acid (EDTA). The samples were immediately centrifuged for 10 minutes (3,000 rpm), and the plasma was stored at -70°C until analysis.

#### Coagulation parameters

Routine laboratory parameters such as prothrombin time (PT), partial thromboplastin time (PTT), antithrombin III (AT III), and fibrinogen were assessed before surgery, four and 24 hours postoperatively, and at discharge. Concentrations of proteins C and S were evaluated preoperatively. Activated protein C resistance was excluded in all patients.

##### Fragment 1+2

Cleavage of fragment 1+2 from prothrombin results in thrombin formation. This fragment remains in the blood circulation long enough to allow quantification of activation of the blood coagulation process. Measurement of F1+2 was performed by enzyme-linked immunosorbent assay (ELISA) (Dade Behring Holding GmbH, Eschborn, Germany) based on capture and labeled antibodies with peroxidase conversion of phenyldiamine-dihydrochloride. The colour formation after conversion was measured at 492 nm. In healthy adults, concentrations range between 0.4 and 1.1 nmol/l.

##### TFPI

TFPI activity can be measured by the catalytic activity of the VIIa/TF complex to activate factor X to Xa. The measurement in EDTA plasma was performed by means of the extent of inhibition of substrate cleavage by factor Xa, which was determined by a colorimetric assay (American Diagnostica Inc., Stamford, CT, USA). Normal range in human plasma has not been generally established yet. Single studies show adult values between 0.83 and 1.14 U/ml [7].

##### TxB2

TxB2 was measured by means of an enzyme immunoassay (Biotrak; Amersham, now part of GE Healthcare, Little Chalfont, Buckinghamshire, UK), based on competition of labeled TxB2 with sample TxB2. In this test, the label was a peroxidase, which converts the substrate tetramethylbenzidine, yielding a yellow colour, which is measured at 450 nm. This assay is very sensitive, being validated to 3.6 pg/ml. Such low concentrations are expected to be found in plasma obtained from healthy volunteers [15].

##### MCP-1

MCP-1 was measured by means of a sandwich ELISA based on a monoclonal capture antibody and a chromogen substrate labeled second antibody (R&D Systems, Inc., Minneapolis, MN, USA). Normal values in adults range from 70 to 300 pg/ml.

### Statistical analysis

Results are expressed as the mean value ± standard error of the mean. Data were analysed with the SPSS for Windows software, version 12 (SPSS GmbH Software, München, Germany). Non-normally distributed variables were analysed by non-parametric tests. Time-dependent variations of biologic variables were analysed by the Wilcoxon test and intergroup comparisons by the Mann-Whitney *U *test. Alpha adjustment for repeated comparisons was performed according to Bonferroni-Holm. The Fisher exact test was used for the analysis of categorical data and the Spearman correlation coefficient for correlation analysis. Probability values less than 0.05 were considered significant.

## Results

### Clinical data

Patients' diagnosis, clinical data, and operation parameters are summarised in Table 1. Children undergoing BV repair were significantly older (*p *< 0.01) but showed similar preoperative oxygen saturation than children undergoing TCPC. CPB time and aortic cross-clamping time, but not duration of cardiocirculatory arrest, were significantly longer in children undergoing BV repair (both *p *< 0.01). There was no difference in minimal oesophageal temperature during CPB in both groups.

Chylothorax occurred in two of the patients and non-chylous pleural or pericardial effusions in 12 children. Nine patients developed transient postoperative arrhythmias. One patient showed multiple organ failure and received peritoneal dialysis. There was no hospital mortality. Postoperative data are given in Table 2.

### Coagulation parameters and anticoagulant treatment at discharge

Levels for proteins C and S were normal in all patients preoperatively, and resistance against active protein C was not seen in any of the patients (data not shown). Values for prothrombin activity, PTT, fibrinogen, and AT III are given in Table 3. AT III level was normal in only four patients: two in the TCPC group and two in the BV group. In all other patients, AT III ranged from 22% to 28% of normal. Seventeen of the 18 patients were treated with heparin starting on day one postoperatively. In contrast to patients after TCPC, patients after BV repair received no anticoagulant treatment at discharge. Nine out of 10 children after TCPC were on anticoagulant treatment at discharge: seven children received coumarin and two patients acetylsalicylic acid. Thus, patients after TCPC showed significantly lower prothrombin activity at discharge (*p *< 0.01). Values for fibrinogen were significantly lower after TCPC than after BV 72 hours postoperatively (*p *< 0.01). Values for partial PT were significantly higher after TCPC than after BV at discharge (*p *< 0.03).

### Coagulation activity

In all children, preoperative levels of F1+2, TxB2, or MCP-1 did not correlate with the age of the patients but TFPI did (Spearman correlation coefficient = -0.68; *p *< 0.05). There was no correlation between the preoperative arterial oxygen saturation and the preoperative coagulation variables. Low AT III concentrations correlated with higher F1+2, shorter PTT, and more postoperative MCP-1. Values for F1+2 did not correlate with TFPI or protein C values. After CPB, lower TFPI concentration correlated with a shorter PT. Significant correlations between coagulation parameters are shown in Table 4.

### Prothrombin fragment 1+2

In all patients, preoperative plasma concentrations of F1+2 were increased in comparison with expected values in adults (4.76 ± 1.39 nmol/l) and raised significantly after heparin administration until 10 minutes after CPB (*p *< 0.01) to reach a maximum value at the end of CPB (115.51 ± 23.75 mol/l). Postoperatively, F1+2 concentrations decreased to reach baseline values from the first postoperative day on.

**Figure 2:**
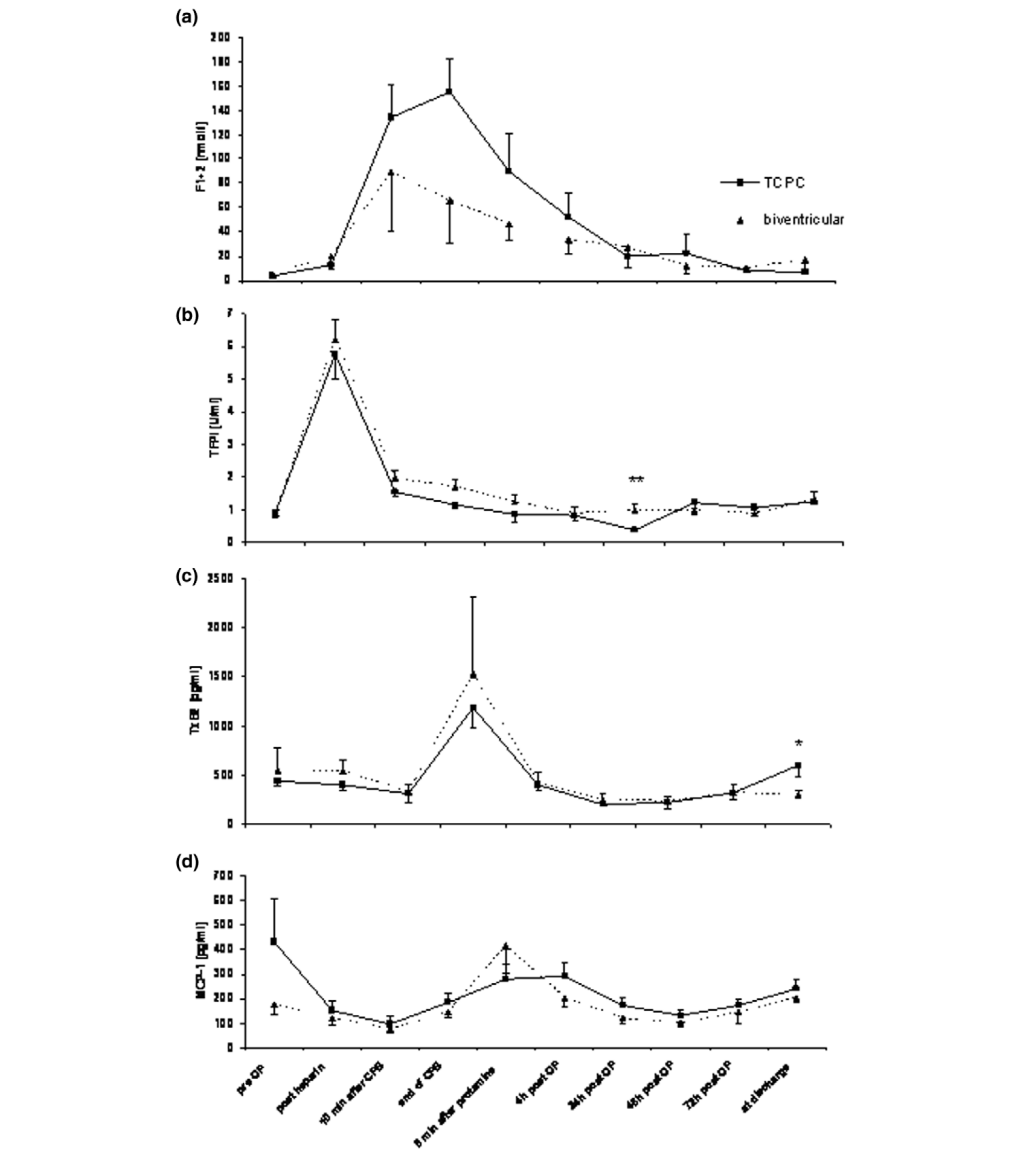
Perioperative course of prothrombin fragment 1 + 2 (F1+2), tissue factor pathway inhibitor (TFPI), thromboxane B_2 _(TxB2), and monocyte chemoattractant protein-1 (MCP-1). **(a) **Time course of F1+2 in both patient groups. F1+2 concentrations increased after heparin, reaching a maximum value at the end of cardiopulmonary bypass (CPB). Postoperatively, F1+2 concentrations showed baseline values. **(b) **Time course of TFPI in both patient groups. TFPI levels increased after heparin administration and decreased from the end of CPB on to reach baseline values. **p < 0.01 comparing both patient groups. **(c) **Time course of TxB2 in both patient groups. TxB2 values decreased at the beginning of CPB and reached maximum values at the end of CPB. In the postoperative course, TxB2 levels were found to be similar to preoperative values. *p < 0.05 comparing both patient groups. **(d) **Time course of MCP-1 in both patient groups. MCP-1 concentrations increased during CPB to reach preoperative values five minutes after protamine administration. TCPC, total cavopulmonary connection.

There was no intergroup difference of F1+2 concentrations at any time point in patients undergoing TCPC as compared with those undergoing BV repair. Figure 2a shows the course of F1+2 before, during, and after CPB for both patient groups.

**Figure 3:**
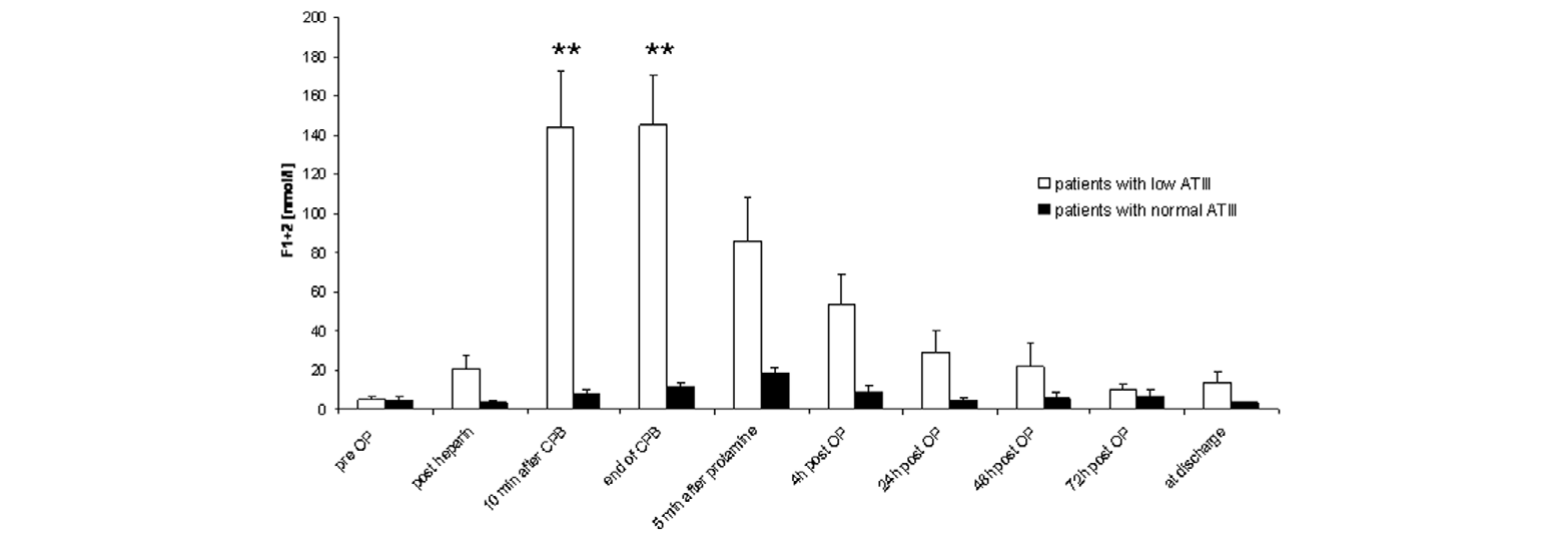
Correlation between antithrombin III (AT III) values and prothrombin fragment 1+2 (F1+2). Patients with normal AT III levels show almost no F1+2 production in the peri- and postoperative courses. During cardiopulmonary bypass (CPB), low AT III correlates with high F1+2: **p < 0.01. post OP, postoperatively; pre OP, preoperatively.

An inverse correlation was found between the concentrations of F1+2 at the end of CPB and five minutes after protamine administration in relation to the minimal oesophageal temperature during CPB (Spearman correlation coefficients were -0.69 [*p *< 0.01] and -0.51 [*p *< 0.05], respectively). There was no correlation between duration of CPB, aortic clamping time, or cardiocirculatory arrest time and F1+2 levels. Four patients in the study had normal AT III concentrations. In those four patients, almost no F1+2 was generated (Figure 3).

### TFPI

Preoperative concentrations of TFPI were normal as compared with values in adults (0.8 ± 0.1 U/l). After heparin administration and before connection to CPB, TFPI levels increased significantly in comparison with preoperative values (5.97 ± 0.48 U/l; *p *< 0.01) as shown in Figure 2b. Ten minutes after CPB, the TFPI levels were still significantly elevated in comparison with preoperative values but decreased from the end of CPB on to reach baseline values. TFPI levels were not different between both patient groups before the operation but were significantly lower in the TCPC group as compared with the BV group 24 hours postoperatively (*p *< 0.01). There was no correlation between duration of CPB, aortic clamping time, or cardiocirculatory arrest time and TFPI levels.

### TxB2

In all patients, preoperative TxB2 values (492.3 ± 110.3 pg/ml) were higher than values observed in normal adults and decreased at the beginning of CPB (321.68 ± 45.77 pg/ml) as shown in Figure 2c. TxB2 values rose significantly 10 minutes after CPB to reach their peak at the end of CPB (1337.0 ± 357.55 pg/ml; *p *< 0.01). Levels decreased significantly again five minutes after protamine administration (*p *< 0.01) and reached preoperative levels 72 hours postoperatively. The concentrations of TxB2 were similar in both patient groups before, during, and after CPB except 72 hours postoperatively. At that time point, the TCPC group showed significantly higher concentrations than the BV group (*p *< 0.05). There was no correlation between duration of CPB, aortic clamping time, or cardiocirculatory arrest time and TxB2 levels.

### MCP-1

In all patients, preoperative MCP-1 values were in the upper normal range for healthy children (288.66 ± 86.73 pg/ml). MCP-1 concentrations decreased significantly 10 minutes after CPB (86.79 ± 18.15 pg/ml; *p *< 0.01) compared with the preoperative values as shown in Figure 2d. MCP-1 concentrations increased during CPB to reach preoperative values five minutes after protamine administration. This was followed by a second significant decrease 48 hours after CPB (119.02 ± 14.68 pg/ml; *p *< 0.01). MCP-1 levels returned to baseline values at the time of discharge in all children. There was no significant difference in MCP-1 levels before, during, and after CPB between both patient groups. There was no correlation between duration of CPB, aortic clamping time, or cardiocirculatory arrest time and MCP-1 levels.

## Discussion

During CPB, accelerated thrombin generation plays a central role in the development of haemostatic abnormalities [1-3,16]. Despite systemic application of heparin during CPB, thrombin activation occurs mainly via the extrinsic coagulation pathway [2,3]. However, the question of whether cardiac surgery with CPB influences the perioperative balance between pro- and anti-thrombotic activity in children with complex cardiac defects has not been addressed so far.

In this study, we report an increased thrombin generation despite liberation of TFPI as an effect of CPB in children undergoing cardiac surgery for complex cyanotic cardiac defect. We could confirm a procoagulant state related to cardiac surgery under CPB, with significantly increased values of F1+2, TxB2, and MCP-1 reaching a maximum at the end of CPB. TFPI was liberated already before initiation of CPB, after heparin administration. Its increase was short-lasting, with levels returning to preoperative values at the end of bypass.

Increased thrombin generation determined by F1+2 levels during cardiac surgery was also shown by others [17,18]. It is generally accepted that thrombin is generated by the contact between blood and the surface of the extracorporeal circuit by initiation of factor X or XII as well as by tissue factor released from the wound and activated monocytes [7,8,19]. We observed an inverse correlation of F1+2 and AT III levels. Low AT III concentrations of approximately 25% will allow more thrombin activity and thus more generation of F1+2.

### Procoagulant state

In our patients, the procoagulant state persisted in the early postoperative period as shown by increased levels of F1+2 up to the first postoperative day. These changes that are due to CPB were not influenced by the type of cardiac surgery performed in our patients [2,3]. The inverse correlation observed between the minimal oesophageal temperature during CPB and F1+2 suggests that hypothermia induces prothrombin cleavage. This goes along with results of other investigations reporting higher thrombin generation and higher platelet aggregation due to deep hypothermia [20,21]. Thrombin and plasmin generation were found to be independent of age in children [4]. Our data confirm this. Therefore, we suggest that age does not have a relevant impact on pro- and anti-thrombotic balance.

In our series, slightly elevated values of F1+2 were still noticed postoperatively and at discharge without any signs of clinical thrombosis. This goes along with results of others showing persistence of increased F1+2 values five days after the Fontan operation [13].

### Balance between pro- and anti-thrombotic activity

Anticoagulant activity of thrombin results in the release of TFPI [2]. In our patients, levels of TFPI increased after heparin administration, reaching their peak value at that time point, according to previous observations [7,8]. Although heparin should reduce the generation and activity of thrombin, thrombin generation as shown by elevated F1+2 was still present in the course of the operation. This observation is confirmed by others [17,18] and is even more present in children compared with adults [22]. In addition, manifest thrombin generation has been shown by measuring thrombin-antithrombin complex, which was also found to be increased in children after CPB compared with adults [22]. Preoperatively, we observed low AT III and short PTT, particularly in the TCPC group. This might explain, in part, the prothrombotic effects in those patients during CPB.

The fact that we detected *in vivo *thrombin generation despite increased levels of TFPI implies that the inhibition of tissue factor-dependent coagulation pathway was not efficient in preventing thrombin generation during CPB [7]. The *in vitro *laboratory PT correlated with TFPI, showing that TFPI as an inhibitor protects against extrinsic clotting. However, TFPI is quickly lost after its release due to heparin administration. Our results suggest that TFPI activity at the end of CPB is insufficient to prevent thrombin generation.

It has been stated that the effect of heparin is inadequate in the paediatric population if detected by ACT measurement [22]. As a consequence, a different monitoring and administration of a higher dosage of heparin during CPB is suggested to reduce thrombin formation [22].

Thrombin is also known as a potent platelet activator. In our study, platelet activation was assessed by measuring TxB2. TxB2 levels reached their peak levels at the end of bypass in our patients. These results go along with other studies reporting that thromboxane values increase and remain elevated during CPB [15,23-25]. The observed correlation between cardiocirculatory arrest time and TxB2 five minutes after protamine administration suggests the importance of the operation technique. The major sources of TxB2 during CPB are thought to be the ischaemic pulmonary tissue and the sequestered platelets [6,24]. The elevated TxB2 values displayed by our patients 72 hours postoperatively could be caused by the contact between blood and the abnormal intracardiac surfaces, increased shear stress, persistent platelet dysfunction due to CPB, or chronic endothelial injury [6,23].

### Interaction between coagulation and inflammation

MCP-1 plays an important role as a mediator between inflammation that is elicited by CPB and coagulation [9,26-28]. MCP-1 upregulation is induced by thrombin-stimulated platelets [29], while MCP-1 in turn induces accumulation of tissue factor [30]. Platelet-monocyte aggregates contribute importantly to thrombosis in vital organs [31]. Increased levels of MCP-1 correlated with a complicated postoperative course after paediatric CPB [32].

Recent studies suggest that thrombin itself is a physiologic mediator of inflammatory events [33-35]. Thrombin receptor activation on leucocytes increases the release of inflammatory cytokines [35]. Furthermore, thrombin contributes to the inflammatory reaction by activating a family of protease-activated receptors, which stimulate cells to express cytokines and chemokines [35]. In our study, this is reflected by a high postoperative MCP-1. This increase seems to be generated during CPB because it is correlated with higher F1+2 and lower AT III. The postoperative increase of MCP-1 might contribute to a higher risk of thromboembolic events in that period. We did not observe any thromboembolic event in our patient group, which might be due to the small number of patients investigated. Long-term evaluations of MCP-1 levels in children after cardiac surgery are mandatory to assess this. An additional important observation of this study is the fact that high levels of MCP-1 were already measured preoperatively and also in the postoperative period until discharge of the patients. Nevertheless, preoperative MCP-1 levels did not correlate with postoperative levels.

Our group has previously described that children with congenital cardiac defects show increased systemic and intramyocardial release of proinflammatory cytokines [36]. Inflammatory cytokines such as interleukin-1 or tumour necrosis factor-α have a prothrombotic effect due to the activation of monocytes and endothelial cells to express tissue factor [34,35]. Children with congenital cardiac defects may therefore have increased prothrombotic state, as suggested by our results showing higher levels of MCP-1 than in healthy children.

### Coagulation and complex congenital heart disease

Coagulation parameter abnormalities that could lead to a higher risk for thromboembolic events such as decreases in protein C, protein S, and plasminogen have been shown after TCPC and, recently, also after partial cavopulmonary connection [37-39]. Recent studies have suggested that, in this latter group of patients scheduled for TCPC, coagulation abnormalities already exist before the operation, as shown by lower concentrations of protein C, factors II, V, VII, and X, plasminogen, and AT III compared with age-matched controls [16].

AT III is known as a crucial parameter for outcome after cardiac operation in adults [40]. However, in children, AT III levels were shown to be lower than in adults, which is also demonstrated in our results [41]. AT III consumption at this stage is suggested. Interestingly, AT III values were already low in our children before the operation. An influence of cyanosis or disturbed liver function might contribute to low AT III levels in these children with complex cyanotic heart disease. In our opinion, the preoperative state might be crucial to a consecutive imbalance in the coagulation system during bypass, leading to thrombin generation. This is supported by the fact that bypass time and the minimal oesophageal temperature did not contribute to F1+2 production during bypass.

As a consequence, in at-risk patients presenting with low AT III levels, AT III replacement could be discussed as a beneficial therapeutic option, but increased risk of bleeding in connection with additional heparin therapy has to be taken in account. It has been shown that AT III replacement in sepsis patients does not lead to a clear benefit; especially in patients co-treated with heparin, the outcome did not improve [42]. Our data show that patients with normal AT III values preoperatively show almost no thrombin generation during the peri- and postoperative course, which might implicate a benefit of AT III substitution in this patient group. At this stage, no data are available in the literature which would justify a routinely applied AT III replacement therapy in children undergoing CPB. A controlled study would be needed to asses the influence of AT III replacement on coagulation disturbances in CPB patients and their outcome.

## Conclusion

Our data show profound coagulation abnormalities occurring during CPB in children with complex congenital cardiac defects undergoing cardiac surgery with an imbalance between pro- and anti-thrombotic activity during CPB. This is shown by the fact that high levels of procoagulant factors as shown by F1+2 are not counterbalanced by anticoagulant factors such as TFPI and AT III. This imbalance is likely to enhance the risk for thromboembolic events during and after cardiac surgery, particularly in patients after univentricular palliation. New studies should clarify whether a therapeutic strategy aimed to enhance anti-thrombotic activity would be beneficial for children undergoing cardiac operations.

## Key messages

• Children with defects who are undergoing cardiac surgery present an imbalance between pro- and anti-thrombotic activity.

• Low AT III levels play an important role in determining coagulation disorders.

## Abbreviations

ACT = activated clotting time; AT III = antithrombin III; BV = biventricular; CPB = cardiopulmonary bypass; EDTA = ethylenediaminetetraacetic acid; ELISA = enzyme-linked immunosorbent assay; F1+2 = prothrombin fragment 1+2; MCP-1 = monocyte chemoattractant protein-1; PT = prothrombin time; PTT = partial thromboplastin time; TCPC = total cavopulmonary connection; TFPI = tissue factor pathway inhibitor; TxB2 = thromboxane B_2_.

## Competing interests

The authors declare that they have no competing interests.

## Authors' contributions

RH performed the analysis of the data and drafted the manuscript. WvO carried out the specific tests for coagulation parameter analysis and participated in the design of the study and interpretation of data. SW collected all clinical and routine laboratory data. KS helped with the statistical analysis. RGG participated in the design of the study. BJM participated in the collection of the data. M-CS participated in the design and coordination of the study and helped to draft the manuscript. All authors read and approved the final manuscript.

## Figures and Tables

**Table 1:** Clinical diagnosis and underlying surgical procedures

Parameter	TCPC group *n*	BV repair group *n*
Underlying diagnosis		
Tricuspid atresia	5	
Pulmonary atresia with or without VSD	3	6
Complex cardiac malformation	2	2
Palliative operations		
Blalock-Taussig shunt	6	5
Glenn operation	3	
Other procedures	2	7
Gender		
Female	5	6
Male	5	2
	
	Mean ± SEM	Mean ± SEM
	
Cardiopulmonary bypass		
Bypass time (minutes)	95 ± 13	217 ± 23
Cardiocirculatory arrest (minutes)	58 ± 2	45 ± 9^a^
Minimal oesophageal temperature (°C)	14.3 ± 0.5	16.6 ± 1.0
Aortic clamping time (minutes)	70 ± 6	131 ± 10
	
	Mean ± SEM	Mean ± SEM
	
Age at operation (months)	34 ± 5	100 ± 19
Weight at operation (kg)	12.4 ± 1.2	23.6 ± 4.1
Preoperative arterial oxygen saturation (percentage)	77 ± 2	86 ± 4
Total number of patients	10	8

^a^Four patients. BV, biventricular; SEM, standard error of the mean; TCPC, total cavopulmonary connection; VSD, ventricular septal defect.

**Table 2:** Postoperative clinical data

Parameter	TCPC group Mean ± SEM	BV repair group Mean ± SEM
Inotropic support (μg/kg per minute)		
Dopamine	4.85 ± 0.09	4.74 ± 0.27
Epinephrine	0.09 ± 0.02	0.08 ± 0.03
Dobutamine	5.15^a^	7.39 ± 1.32^b^
Sodium nitroprusside	0.98 ± 0.07	0.81 ± 0.21
Duration of mechanical ventilation (days)	5.3 ± 1.2	2.0 ± 0.8
Duration of intensive care treatment (days)	12.9 ± 2.5	3.6 ± 0.8
	
Blood transfusions up to four hours postoperatively (ml/kg)	Mean ± SEM	Mean ± SEM
	
Erythrocytes	8.9 ± 1.3	11.6 ± 5.1
Human albumin 5%	12.0 ± 4.0	11.0 ± 4.3
Fresh frozen plasma	11.9 ± 0.6	8.3 ± 1.1
CPB blood	16.2 ± 6.1	8.3 ± 1.4
	
Blood transfusions 4 to 24 hours postoperatively (ml/kg)	Mean ± SEM	Mean ± SEM
	
Erythrocytes	90.0^a^	9.0 ± 1.7
Human albumin 5%	22.4 ± 3.7	7.2 ± 0.4
Fresh frozen plasma	11.7 ± 1.3	
CPB blood		11.2^a^

^a^One patient; ^b^three patients. BV, biventricular; CPB, cardiopulmonary bypass; SEM, standard error of the mean; TCPC, total cavopulmonary connection.

**Table 3:** Course of coagulation parameters

Coagulation parameter	TCPC group Mean ± SEM				BV repair group Mean ± SEM			
Time point	PT (percentage)	PTT (seconds)	Fibrinogen (mg/dl)	AT III (percentage)	PT (percentage)	PTT (seconds)	Fibrinogen (mg/dl)	AT III (percentage)

Preoperative	92.3 ± 2.5	32.2 ± 1.0	2.8 ± 0.2	43.8 ± 12.6	89.0 ± 8.0	36.0 ± 1.7	3.0 ± 0.2	46.9 ± 13.7
4 hours postoperative	62.3 ± 3.9	47.7 ± 5.6	1.5 ± 0.1	20.4 ± 4.1	70.9 ± 3.4	37.8 ± 1.7	1.7 ± 0.3	27.4 ± 6.9
24 hours postoperative	51.4 ± 6.2	39.5 ± 35.9	1.9 ± 0.2^a^	20.9 ± 4.7	62.8 ± 4.7	33.9 ± 2.4	2.9 ± 0.4^a^	38.5 ± 13.4
72 hours postoperative	75.5 ± 6.7	45.3 ± 11.9	2.8 ± 0.3^a^	37.9 ± 14.2	84.0 ± 8.5	33.5 ± 1.9	4.1 ± 0.5^a^	48.2 ± 17.7
At discharge	47.0 ± 8.5^a^	37.0 ± 1.7^a^	nd	nd	95.3 ± 1.8^a^	31.3 ± 1.6^a^	nd	nd

^a^Significant difference (*p *≤ 0.05) of values between the two groups. AT III, antithrombin III; BV, biventricular; nd, not diagnosed; PT, prothrombin time; PTT, partial thromboplastin time; SEM, standard error of the mean; TCPC, total cavopulmonary connection.

**Table 4:** Correlations of coagulation parameters

	AT III (preoperative)	AT III (four hours postoperative)	MCP-1 (four hours postoperative)	PT (four hours postoperative)	PTT (four hours postoperative)
F1+2 (end of CPB)	Negative (*p *< 0.05)	Negative (*p *< 0.01)	Positive (*p *< 0.01)		
F1+2 (five minutes after protamine)	Negative (*p *< 0.05)	Negative (*p *< 0.01)			
F1+2 (four hours postoperative)	Negative (*p *< 0.01)			Positive (*p *< 0.01)	
MCP-1 (four hours postoperative)	Negative (*p *< 0.05)	Negative (*p *< 0.01)			
TFPI (five minutes after protamin)				Positive (*p *< 0.05)	Negative (*p *< 0.05)

AT III, antithrombin III; BV, biventricular; CPB, cardiopulmonary bypass; F1+2, prothrombin fragment 1+2; MCP-1, monocyte chemoattractant protein-1; PT, prothrombin time; PTT, partial thromboplastin time; TFPI, tissue factor pathway inhibitor.
